# Empagliflozin prevents neointima formation by impairing smooth muscle cell proliferation and accelerating endothelial regeneration

**DOI:** 10.3389/fcvm.2022.956041

**Published:** 2022-08-09

**Authors:** Jochen Dutzmann, Lena Marie Bode, Katrin Kalies, Laura Korte, Kai Knöpp, Frederik Julius Kloss, Mirja Sirisko, Claudia Pilowski, Susanne Koch, Heiko Schenk, Jan-Marcus Daniel, Johann Bauersachs, Daniel G. Sedding

**Affiliations:** ^1^Mid-German Heart Center, Division of Cardiology, Angiology and Intensive Medical Care, University Hospital Halle, Martin-Luther-University Halle-Wittenberg, Halle (Saale), Germany; ^2^Department of Cardiology and Angiology, Hannover Medical School, Hanover, Germany; ^3^Department of Nephrology and Hypertension, Hannover Medical School, Hanover, Germany

**Keywords:** vascular remodeling, neointima formation, restenosis, SGLT2, diabetes

## Abstract

**Background:**

Empagliflozin, an inhibitor of the sodium glucose co-transporter 2 (SGLT2) and developed as an anti-diabetic agent exerts additional beneficial effects on heart failure outcomes. However, the effect of empagliflozin on vascular cell function and vascular remodeling processes remains largely elusive.

**Methods/Results:**

Immunocytochemistry and immunoblotting revealed SGLT2 to be expressed in human smooth muscle (SMC) and endothelial cells (EC) as well as in murine femoral arteries. *In vitro*, empagliflozin reduced serum-induced proliferation and migration of human diabetic and non-diabetic SMCs in a dose-dependent manner. In contrast, empagliflozin significantly increased the cell count and migration capacity of human diabetic ECs, but not of human non-diabetic ECs. *In vivo*, application of empagliflozin resulted in a reduced number of proliferating neointimal cells in response to femoral artery wire-injury in C57BL/6J mice and prevented neointima formation. Comparable effects were observed in a streptozocin-induced diabetic model of apolipoprotein E^–/–^ mice. Conclusive to the *in vitro*-results, re-endothelialization was not significantly affected in C57BL/6 mice, but improved in diabetic mice after treatment with empagliflozin assessed by Evan’s Blue staining 3 days after electric denudation of the carotid artery. Ribonucleic acid (RNA) sequencing (RNA-seq) of human SMCs identified the vasoactive peptide apelin to be decisively regulated in response to empagliflozin treatment. Recombinant apelin mimicked the *in vitro*-effects of empagliflozin in ECs and SMCs.

**Conclusion:**

Empagliflozin significantly reduces serum-induced proliferation and migration of SMCs *in vitro* and prevents neointima formation *in vivo*, while augmenting EC proliferation *in vitro* and re-endothelialization *in vivo* after vascular injury. These data document the functional impact of empagliflozin on vascular human SMCs and ECs and vascular remodeling in mice for the first time.

## Introduction

Type 2 diabetes mellitus (T2DM) is a major risk factor for the development of in-stent restenosis after revascularization procedures in coronary artery disease. Despite the pioneering advance of using drug-eluting stents in revascularization therapy, in-stent-restenosis still occurs in approximately 1 out of 10 patients undergoing coronary artery stent implantation and patients with T2DM face a considerable higher risk of developing restenosis compared to patients with normoglycemic conditions according to the National Cardiovascular Data Registry database (1). Hallmarks of neointima formation are a disruption of endothelial integrity following revascularization and stent expansion in the vascular wall, accompanied by an inflammatory response, and consecutive stimulation of SMC proliferation and migration from the media toward the intimal layer (2, 3).

Sodium glucose co-transporter 2 (SGLT-2) inhibitors, gliflozins, initially FDA- and EMA-approved for the treatment of diabetes mellitus, have recently been recommended as first-line treatment for heart failure with reduced ejection fraction by several international cardiac societies (4–6). In fact, empagliflozin prevented hospitalizations for heart failure and cardiovascular death compared to placebo in T2DM patients with established coronary artery disease as shown in the EMPA-REG OUTCOME trial in 2015 (7). The ensuing EMPEROR-REDUCED and EMPEROR-PRESERVED trials confirmed these results regardless of the presence or absence of diabetes in 2020 and 2021 (8, 9). Intriguingly, the cardio-protective effects of empagliflozin were already seen at very early time points after randomization in both trials.

Not surprisingly, these results triggered a lively discussion on the pathophysiological and molecular mechanisms contributing to the beneficial effects on heart failure outcomes (10–13). SGLT-2 has not been detected in cardiomyocytes and the heart yet and sole normalization of blood glucose levels takes longer periods of time to exert cardiovascular benefits. Various reports suggested direct effects of SGLT-2 inhibition on vascular function (14). Yet, actual proof for any of these mechanisms is still lacking.

Here, we show direct and immediate effects on smooth muscle cell and endothelial cell proliferation and migration *in vitro* and neointimal lesion formation *in vivo*. These data suggest that direct vascular effects potentially contribute to the early cardio-protective effects of empagliflozin seen.

## Materials and methods

### Cell culture

Diabetic and non-diabetic human coronary artery smooth muscle cells (SMC) and human coronary artery endothelial cells (EC) were purchased from Lonza (Cologne, Germany). Cells between passages 3 and 6 were used for all experiments and cultured in optimized growth medium according to the supplier’s protocols.

### Functional *in vitro* assays

Total cell count was determined by cleavage of WST-1 to formazan (Cell Proliferation Reagent WST-1, Roche, Basel, Switzerland) and colorimetric measurement following manufacturer’s instructions and as previously described (15). For siRNA-mediated knockdown cells were transfected with siRNA (4390843 and s12955, Ambion, Thermo Scientific, Rockford, IL, United States, and HuSR305853, OriGene Technologies, Rockville, MD, United States) according to the manufacturer’s instructions and as indicated before serum-starvation followed by stimulation with 5% fetal calf serum (FCS). In brief, siRNAs were mixed with Opti-MEM^®^ I medium (Thermo Scientific) and incubated with Lipofectamine^®^ 2000 (Thermo Scientific) for 5 min. The mixture was added to the cells and incubated for 24 h. For inhibitor studies, indicated concentrations of empagliflozin were added to FCS. Recombinant full length apelin used has been the 77 amino acids preprotein, that is later processed to active apelin (ab152927, Abcam, Cambridge, United Kingdom).

Migration capacity was determined *via* a scratch-wound assay. The assay was performed in 24-well plates with cells seeded to confluence. After cell adherence to the well bottom a scratch wound was done with a pipette tip. Images were taken every 30 min over a period of 24 hwith a Cytation 1 (Biotek, Winooski, VT, United States). Scratch-area and cell-area were calculated with the Gen5 software (Biotek) for every image.

Apoptosis was determined by analysis of histone-associated deoxyribonucleic acid (DNA) fragments in the cytoplasm of apoptotic cells (REF 11544675001, Roche). Briefly, cells were seeded in a 96-well plate to confluence. Cells were incubated as indicated for 24 h, subsequently suspended in a sample solution, and added to the prepared microplate modules according to the manufacturer’s instructions. Afterward, absorbance was measured using a colorimetric ELISA reader and apoptosis was assessed as specific enrichment of mono- and oligonucleosomes released into the cytoplasm according to the manufacturer’s instructions.

### Preparation of cellular lysates and immunoblot analysis

For cellular lysis, cells were washed twice in phosphate buffered saline and lysed in protein lysis buffer. Lysates were incubated on ice for 30 min and subsequently centrifuged at 4°C and 13000 rpm for 15 min. Protein concentration of the supernatant has been determined using a Bradford protein assay (Bio-Rad). After addition of sample buffer, dithiothreitol, and H_2_O, the protein has been denatured at 95°C for 5 min. Protein mix was run on commercial precast 4–12% Bis-Tris gels (NuPAGE^®^ SDS-PAGE Gel System, LifeTechnologies, Carlsbad, CA, United States) and transferred onto a nitrocellulose membrane by a commercial wet/tank blotting system (*Trans-*Blot^®^, Bio-Rad Laboratories, Hercules, CA, United States). After blocking with 5% milk, the blots were incubated with the primary antibody (anti-SGLT2, ab37296, Abcam or anti-GAPDH, sc-32233, Santa Cruz Biotechnology, Dallas, TX, United States) overnight at 4°C. The proteins were then detected by enhanced chemiluminescence (Pierce ECL Plus, Thermo Scientific) after labeling with a horseradish peroxidase-labeled secondary antibody according to the manufacturer’s instructions (sc-2056, sc-2314, or sc-2004, Santa Cruz). Densitometric analysis was performed with ImageJ 1.49v.

### Ribonucleic acid isolation, reverse transcription, and quantitative real-time polymerase chain reaction

Isolation of ribonucleic acid (RNA) from cells was performed using the RNeasy mini kit (Qiagen, Hilden, Germany) and obtained RNA was reverse transcribed with the High-Capacity RNA-to-cDNA™ kit (Applied Biosystems, Foster City, CA, United States) according to manufacturer’s instructions and as previously described (16).

Quantitative real-time polymerase chain reaction (PCR) was performed using SYBR green^®^ master mix with the respective primers and the CFX96 Touch™ Real Time PCR Detection System (both: Bio-Rad Laboratories) as previously described (16). All analyses were performed in triplicate and either the DNA template or the reverse transcriptase was omitted for control reactions. Fold change expression levels were quantified and normalized to the geometric mean of three reference genes with the highest expression stability by use of the 2^–ΔΔ^ *^Ct^* relative quantification method. Primer sequences were as follows: apelin (VHPS-447, Biomol, Hamburg, Germany), glyceraldehyde-3-phosphate dehydrogenase (GAPDH) fwd 5′-TGCACCACCAACTGCTTAGC-3′, rev 5′-GGCATGGACTGTGGTCATGAG-3′; F-box protein 7 (FBXO7) fwd 5′-GCTCGCACCTGAGGCAGTCC-3′, rev 5′-GTCTCTTCATCTCCAGTGAGGGG-3′; actin B fwd 5′-CCTCGCCTTTGCCGATCCG-3′, rev 5′-CGACGAGCGC GGCGATATCATC-3′.

### Animals

All animal experiment procedures complied with the Directive 2010/63/EU of the European Parliament and local ethical guidelines. All procedures had been approved by the Lower Saxony’s institutional committee for animal research (Niedersächsisches Landesamt für Verbraucherschutz und Lebensmittelsicherheit, approval reference number 16/2070) prior to the start of the experiments. All experiments were performed on at least 8-week-old adult male C57BL/6 mice purchased from Charles River (Sulzfeld, Germany). Sample size has been calculated by the Hannover Medical School Institute of Biometrics using nQuery Advisor 7.0. Investigators have been blinded at all stages of the experiments. Reporting in the manuscript follows the recommendations in the ARRIVE guidelines (17).

*Apoe*^*TM* 1*Unc*^ mice were treated with daily intraperitoneal injections of at least 50 mg kg^–1^d^–1^ streptozotozin (Stz; 1.25 mg/200 μl citrate buffer) until serum glucose concentrations > 200 mg/dl, but for at least 5 days (18). Stz-treated mice with serum glucose > 200 mg/dl were classified as diabetic. Empagliflozin was added to mouse chow to deliver 10 mg kg^–1^ d^–1^ for 7 days (0.25 mg daily per animal estimating a mouse weight of 25–30 g and ingestion of 5 g rodent chow daily). Control mice got a standard diet. Serum and urine glucose measurement was performed to ensure the efficacy of Stz- and empagliflozin-treatment. In addition, body weight and serum cholesterol concentrations have been determined (Supplementary Figure I).

### Vascular injury models

#### Mouse carotid artery model of re-endothelialization

Electric de-endothelialization of the carotid artery was performed at day 7 of empagliflozin treatment following previously described protocols (19). Mice were anesthetized using a singular intraperitoneal injection of ketamine hydrochloride (100 mg/kg body weight; Anesketin, Albrecht, Germany) and xylazine (16 mg/kg body weight Rompun^®^ 2%, Bayer Health Care, Leverkusen, Germany). After preparation of the left common carotid artery through ventral middle line neck incision, electric de-endothelialization was carried out with a bipolar microregulator (ICC50, ERBE-Elektromedizin, Tübingen, Germany) at a length of 5 mm below the carotid bifurcation (2 W for 2 s). Three days after electrical injury of the left carotid artery, mice were anesthetized, 50 μl of 5% Evan’s blue dye (Sigma-Aldrich, Darmstadt, Germany) was injected into the tail vein, and mice were sacrificed 5 min after Evan’s blue dye injection by cervical dislocation and perfused with phosphate buffered saline. The left carotid artery was carefully dissected and reendothelialization was assessed by en face staining (Eclipse Ni-E microscope, Nikon, Japan). Re-endothelialization was calculated as the difference between the length of the blue-stained area and the initially injured area, using computer-assisted morphometric analysis (ImageJ 1.48 software, National Institutes of Health, United States).

#### Mouse femoral artery injury model of neointima formation

Wire-induced injury of the femoral artery was performed at day 7 of empagliflozin treatment as previously described (19). In brief, mice were anesthetized as described earlier. A straight spring wire (0.38 mm in diameter, Cook Medical, Bloomington, IN, United States) was then inserted through the profunda femoris artery up to 1 cm into the femoral artery and left in place for 1 min to achieve an adequate wire-induced vessel injury. After removal of the wire, the profunda femoris artery was ligated (7-0 Prolene, Ethicon, Johnson & Johnson, Norderstedt, Germany) and perfusion of the dilated femoral artery was re-established. Mice were sacrificed at 10 or 21 days by cervical dislocation. The femoral artery was carefully harvested and embedded in Tissue-Tek OCT compound medium (Sakura Finetek Europe B.V., Staufen im Breisgau, Germany). Then, the arteries were snap-frozen and stored at –80°C until sectioning.

### Morphometry

After harvesting, the dilated femoral arteries at the indicated time-points following injury, vessels were sliced in 6 μm serial sections and Verhoeff–van Gieson staining (Carl Roth, Karlsruhe, Germany) was performed for six cross-sections from regular intervals throughout each artery. ImageJ 1.48 software was used to calculate circumference of external elastic lamina, internal elastic lamina and lumen as well as medial and neointimal area.

### Immunofluorescence

Samples were incubated with antibodies targeting α-SMA (C6198, Sigma-Aldrich), Ki-67 (ab15580, Abcam), or SGLT-2 (ab37296, Abcam). After incubation with primary antibodies, samples were marked with Alexa 488- or 546-coupled secondary antibodies (Life Technologies, Carlsbad, CA, United States) and counterstained with nuclear 4.6-diamidino-2-phenylindole (Immunoselect Antifading Mounting Medium DAPI, Dianova, Germany). Matching species- and isotype control antibodies were used for negative controls (Santa Cruz). For bright-field and immunofluorescence microscopy an Eclipse NI-E microscope (Nikon Instruments Europe, Netherlands), adequate filter blocks and image processing software were used (NIS Elements AR 4.20.01, Nikon Instruments Europe).

### Ribonucleic acid sequencing and analysis

#### Ribonucleic acid isolation

Ribonucleic acid was isolated as described above. RNA concentrations were measured using Nanodrop 2000c (Thermo Scientific) and Agilent 2100 Bioanalyzer (Agilent Technologies, Santa Clara, CA, United States) according to the manufacturer’s instructions.

#### Ribonucleic acid sequencing library construction

Ribonucleic acid sequencing was conducted by Novogene (Novogene, Cambridge, United Kingdom). In brief, mRNA was enriched using oligo(dT) beads. The mRNA was then fragmented randomly in fragmentation buffer, followed by cDNA synthesis using random hexamers and reverse transcriptase. After first-strand synthesis, a custom second-strand synthesis buffer (Illumina, San Diego, CA, United States) was added with dNTPs, RNase H and Escherichia coli polymerase I to generate the second strand by nick-translation. The final cDNA library was ready after a round of purification, terminal repair, A-tailing, ligation of sequencing adapters, size selection and PCR enrichment. Library concentration was then quantified using a Qubit 2.0 fluorometer (Life Technologies) and diluted to 1 ng/μl before checking insert size on an Agilent 2100 and quantifying to greater accuracy by quantitative PCR (qPCR) (library activity > 2 nM). Libraries were finally fed into HiSeq machines (Illumina) according to activities and expected data volume.

#### Ribonucleic acid sequencing processing

The original raw data was transformed to Sequenced Reads by base calling. Raw data was recorded in a FASTQ file, which contains sequence information (reads) and corresponding sequencing quality information. RNA-seq reads were then aligned to the Ensembl reference genome using the TopHat2 algorithm. Raw count data per gene was calculated using HTSeq software. The raw count matrix was then used by DESeq to quantify gene expression level as normalized counts. Transcripts with an adjusted *P* < 0.05 were considered differentially expressed. Data were analyzed with the Gene Set Enrichment Analysis (GSEA) software, whereas C5 gene ontology gene sets were obtained from MSigDB.

Ribonucleic acid-sequencing data have been deposited in the GEO repository (GEO accession number: GSE189538).

### Statistical analysis

Data was stored and analyzed on personal computers using Microsoft Excel (Microsoft Corporation, Redmond, WA, United States) and GraphPad Prism 8 (GraphPad Software Inc., La Jolla, CA, United States). Study groups were compared using Mann–Whitney *U* test, Kruskal–Wallis *H* test followed by multiple comparisons using Dunn’s method, or ordinary 1 way ANOVA followed by pairwise multiple comparisons using the Tukey method depending on normality, the number of groups, and affecting factors. The two one-sided test (TOST) procedure was used to test equivalence. All data are represented as the mean ± standard error of the mean (SEM). A probability value < 0.05 was considered statistically significant for all comparisons.

## Results

### Sodium glucose co-transporter 2 is expressed in smooth muscles, endothelial cells, and the vascular wall

**FIGURE 1:**
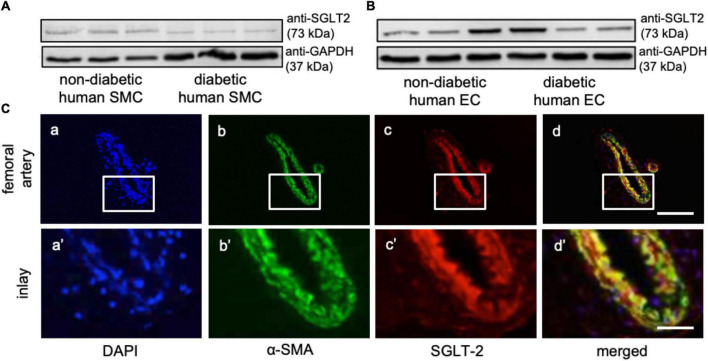
Sodium glucose co-transporter 2 (SGLT2) is expressed in human vascular cells and the murine femoral artery. **(A,B)** Western blot analysis for SGLT2 protein expression and glyceraldehyde-3-phosphate dehydrogenase (GAPDH) chosen as loading control in non-diabetic and diabetic human smooth muscle cells (SMCs, *n* = 3, **A**) and endothelial cells (ECs, *n* = 3, **B**). Full-length blots are presented in Supplementary Figure II. **(C)** Vascular SGLT2 expression in the native murine femoral artery as determined by immunohistochemical co-staining of α-smooth muscle actin (α-SMA, green) with SGLT-2 (red), and DAPI (blue; *n* = 6, upper scale bar 100 μm, lower scale bar 25 μm).

Western-blot analysis revealed robust protein expression levels of SGLT-2 in human SMCs and ECs of non-diabetic as well as diabetic donors (*n* = 3, Figures 1A,B, full-length blots in Supplementary Figure II). However, SGLT-2 protein expression was substantially less compared to human embryonic kidney cells (data not shown). *In vivo*, immunofluorescence analysis of native murine femoral artery specimens confirmed vascular SGLT-2 expression using co-labeling of α-SMA (*n* = 6, Figure 1C, upper scale bar 100 μm, lower scale bar 25 μm).

### Empagliflozin improves vascular cell function *in vitro*

**FIGURE 2:**
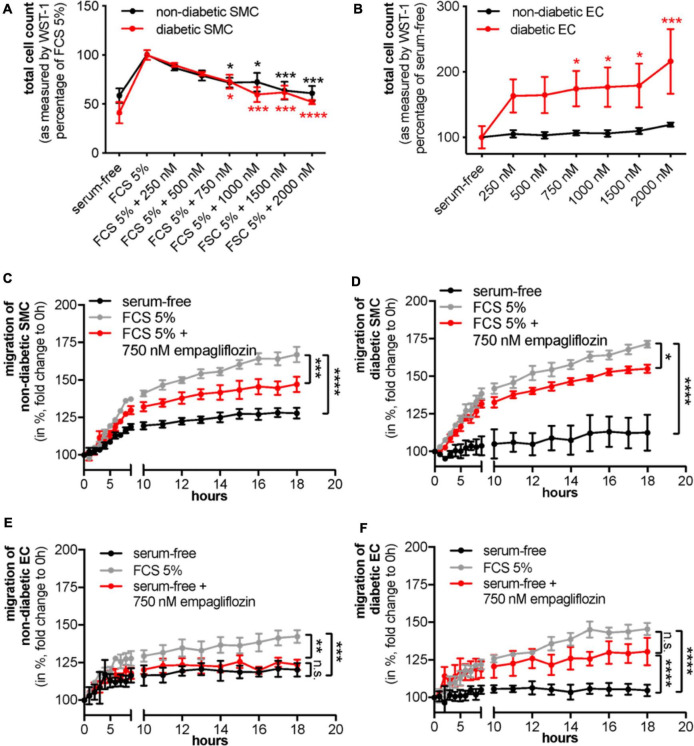
Empagliflozin improves diabetic endothelial cell (EC) function and prevents diabetic and non-diabetic smooth muscle cell (SMC) proliferation and migration. **(A,B)** Total cell count assessment of non-diabetic (black) and diabetic (red) SMCs **(A)** stimulated with 5% fetal calf serum (FCS) and indicated concentrations of empagliflozin and ECs with serum-free medium and indicated concentrations of empagliflozin **(B)** assessed by WST-1 cleavage to formazan at 24 h. **(C–F)** Determination of cell migration of non-diabetic **(C)** and diabetic **(D)** SMCs in serum free media (black), treated with 5% FCS (gray), and FCS 5% supplemented with 750 nM empagliflozin (red) as well as of non-diabetic **(E)** and diabetic ECs **(F)** in serum-free media (black), treated with 5% FCS (gray), and serum-free media supplemented with 750 nM empagliflozin (red) assessed by cell coverage at indicated time points in a scratch-wound assay (**A–F**: *n* = 3, *n.s.*, not significant, *P < 0.05, **P < 0.01, ***P < 0.001, ****P < 0.0001).

To investigate the impact of empagliflozin on vascular cell function *in vitro*, non-diabetic and diabetic coronary artery cells were incubated with empagliflozin. After 24 h, we detected a dose-dependent reduction of the number of non-diabetic as well as diabetic SMCs in response to treatment with empagliflozin as assessed by WST-1 cleavage to formazan (non-diabetic SMCs: FCS 5% 100 ± 2.178% vs. FCS 5% + 750 nM empagliflozin 71.859 ± 4.821%, *n* = 3, *P < 0.05; diabetic SMCs: FCS 5% 100 ± 4.950% vs. FCS 5% + 750 nM empagliflozin 72.685 ± 7.016%, *n* = 3, *P < 0.05; Figure 2A). Reduction in diabetic SMC count upon empagliflozin was potentially partially dependent on SGLT-2 expression, as SGLT-2 knockdown reduced the increase in total cell count as well upon FCS 5% (Supplementary Figure III). The effect was rather cytostatic than cytotoxic as empagliflozin did not induce apoptosis in a cell death detection assay based on the analysis of histone-associated DNA fragments (Supplementary Figure IV). In contrast, just the number of diabetic ECs increased in response to empagliflozin, while the number of non-diabetic ECs remained unaffected (non-diabetic ECs: serum-free 100 ± 3.57% vs. serum-free + 750 nM empagliflozin 106.737 ± 3.608%, *n* = 3, *P* = 0.9997; diabetic ECs: serum-free 100 ± 16.793% vs. serum-free + 750 nM empagliflozin 174.313 ± 26.914%, *n* = 3, *P < 0.05; Figure 2B). Minimum required dosage of empagliflozin to significantly prevent the FCS-dependent increase in vascular cell count was determined at 750 nM in both SMCs and diabetic ECs, a dose that was used for all future *in vitro* experiments. Conclusive to the effects on vascular cell count, empagliflozin impaired the migrational capacity of non-diabetic and diabetic SMCs determined *via* a scratch-wound assay (cell coverage at 18 h compared to 0 h following stimulation; non-diabetic SMCs: serum-free 127.83 ± 3.51% vs. FCS 5% 166.91 ± 5.14% vs. FCS 5% + 750 nM empagliflozin 147.12 ± 5.197%, *n* = 3, ^***^P < 0.001, Figure 2C; diabetic SMCs: serum-free 112.45 ± 11.94% vs. FCS 5% 171.31 ± 2.23% vs. FCS 5% + 750 nM empagliflozin 155.01 ± 0.54%, *n* = 3, *P < 0.05; Figure 2D). It furthermore improved the migrational capacity of diabetic, but not of non-diabetic ECs (cell coverage at 18 h following stimulation; non-diabetic ECs: serum-free 120.91 ± 4.48% vs. FCS 5% 142.33 ± 4.33% vs. serum-free + 750 nM empagliflozin 123.47 ± 3.41%, *n* = 3, *P* = 0.2908, Figure 2E; diabetic ECs: serum-free 104.59 ± 3.87% vs. FCS 5% 145.39 ± 4.08% vs. serum-free + 750 nM empagliflozin 130.45 ± 9.20%, *n* = 3, ^****^P < 0.0001; Figure 2F).

### Empagliflozin accelerates re-endothelialization and prevents neointimal lesion formation *in vivo*

**FIGURE 3:**
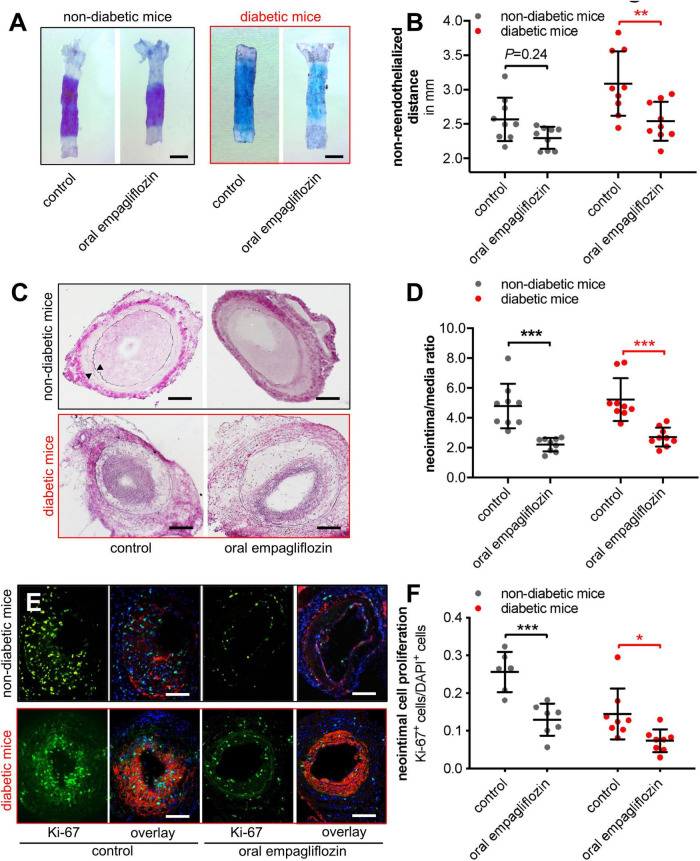
Empagliflozin improves vascular function in mice. **(A,B)** Evan’s blue staining at 3 days after electrical injury of the murine carotid artery in non-diabetic and diabetic mice treated with empagliflozin or standard diet (**A**, scale bar 1 mm) and quantification of non-re-endothelialized distance (**B**, *n* = 9, **P < 0.01). **(C,D)** Verhoeff–Van Gieson staining 21 days following wire-induced injury of non-diabetic and diabetic murine femoral arteries (**C**, scale bar 100 μm, arrowheads mark internal and external elastical laminae) and quantification of neointima/media ratio (**D**, *n* = 9, ***P < 0.0001). **(E,F)** Neointimal cell proliferation as determined by co-staining for Ki-67 (green), of α-smooth muscle actin (red), and DAPI (blue) at 10 days following wire-induced injury of non-diabetic and diabetic murine femoral arteries (**E**, scale bar 100 μm) and quantification (**F**, *n* = 6–8, *P < 0.05, ***P < 0.001).

To study the *in vivo* effects of empagliflozin, we first determined the expanse of re-endothelialization 3 days following electrical de-endothelialization of the carotid artery in non-diabetic or diabetic C57BL/6 mice orally treated with empagliflozin or standard chow. In line with the observed effects *in vitro*, empagliflozin accelerates the re-endothelialization process in diabetic mice *in vivo*, but not in non-diabetic mice (non-re-endothelialized distance; non-diabetic mice: control 2.569 ± 0.105 mm vs. empagliflozin 2.298 ± 0.054 mm, *P* = 0.24; diabetic mice: control 3.088 ± 0.157 mm vs. empagliflozin 2.540 ± 0.094 mm, *n* = 9, ^**^P < 0.01, Figures 3A,B, scale bar 1 mm). Notably, re-endothelialization was significantly compromised in diabetic mice compared to non-diabetic mice (*P* = 0.007) and empagliflozin took the re-endothelialization capacity back to non-diabetic levels as assessed by TOST (pre-set equivalence margin δ = 0.30 mm; 90% confidence interval for the difference between means (–0.22, 0.27), *P* < 0.05).

Oral treatment with empagliflozin impaired neointima formation 21 days after wire-induced injury of the murine femoral artery, conclusive to the aforementioned results in diabetic mice, but also in non-diabetic mice (neointima/media ratio; non-diabetic mice: control 4.79 ± 1.40 vs. empagliflozin 2.20 ± 0.42, *n* = 9, ^***^P < 0.001; diabetic mice: control 5.22 ± 1.35 vs. empagliflozin 2.71 ± 0.60, *n* = 9, ^***^P < 0.001, Figures 3C,D, scale bar 50 μm). Neointimal and medial cell proliferation was prevented by empagliflozin in both non-diabetic and diabetic mice, again in coherence with the effect of empagliflozin on SMC cell number *in vitro* (Ki-67^+^ cells/DAPI^+^ cells; non-diabetic mice: control 0.25 ± 0.0218% vs. empagliflozin 0.13 ± 0.0161, *n* = 6–7, ^***^P < 0.001; diabetic mice: control 0.16 ± 0.0256% vs. empagliflozin 0.07 ± 0.0122, *n* = 8, *P < 0.05, Figures 3E,F, scale bar 50 μm).

### Empagliflozin increases apelin expression in smooth muscles and endothelial cells

**FIGURE 4:**
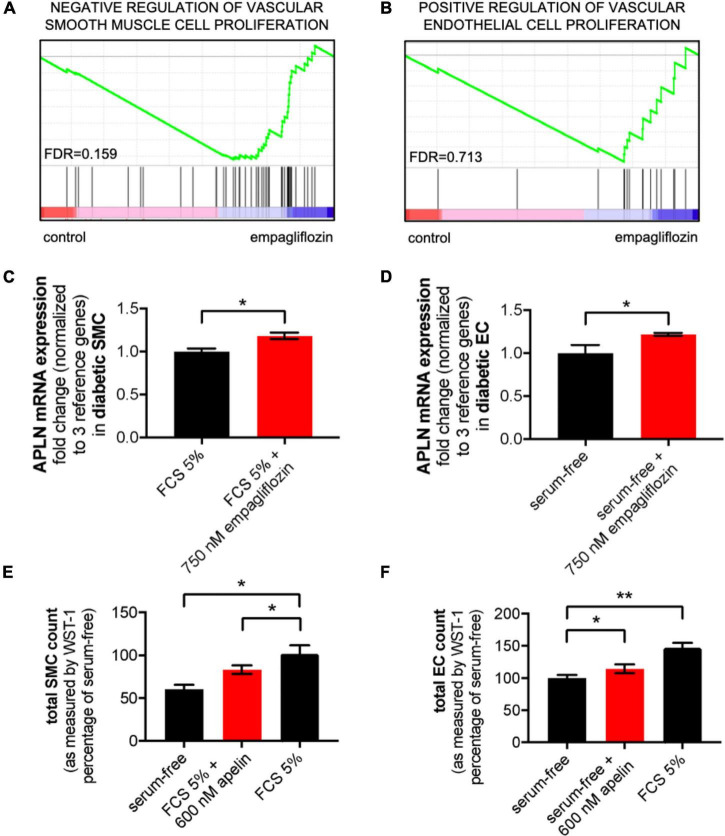
Apelin is upregulated by empagliflozin, prevents smooth muscle cell (SMC) proliferation and augments endothelial cell (EC) proliferation. **(A,B)** Two out of 20 most regulated gene sets from RNA-sequencing data of human diabetic coronary artery SMCs (*n* = 3). **(C,D)** Apelin mRNA expression as assessed by qRT-PCR in diabetic SMCs incubated with 5% fetal calf serum (FCS) with and without 750 nM empagliflozin (**C**, *n* = 3, *P < 0.05) and in diabetic ECs incubated with serum-free medium with and without 750 nM empagliflozin (**D**, *n* = 3, *P < 0.05). **(E,F)** Total cell count assessment of diabetic SMCs **(E)** stimulated with 5% FCS with or without 600 nM recombinant apelin and ECs with serum-free medium with or without 600 nM recombinant apelin **(F)** assessed by WST-1 cleavage to formazan at 24 h (*n* = 3, *P < 0.05, **P < 0.01).

In order to elucidate potential mechanisms of empagliflozin-mediated vascular effects, we performed differential RNA expression analysis on SMCs of diabetic donors by RNA-sequencing (Supplementary Figure V). Consistent with our earlier findings, gene set enrichment analysis revealed *“Negative Regulation of Vascular Smooth Muscle Cell Proliferation”* and *“Positive Regulation of Vascular Endothelial Cell Proliferation”* between the 20 most significantly regulated gene sets (*n* = 3, Figures 4A,B). Though, the last-mentioned was not significant after adjustment for the false discovery rate (FDR; FDR = 0.713). Notably, both gene sets overlapped by the vasoactive peptide apelin.

Quantitative real-time polymerase chain reaction validated significant upregulation of apelin in response to empagliflozin in both diabetic SMCs and diabetic ECs 24 h following stimulation (*n* = 3, Figures 4C,D). To investigate effects of apelin on vasculature, we treated diabetic human smooth muscle cells stimulated with FCS 5% and serum-starved endothelial cells with recombinant apelin. Apelin prevented the FCS-induced increase in SMC count whereas it augmented the number of ECs as assessed by WST-1 cleavage to formazan (diabetic SMCs: FCS 5% 100 ± 9.752% vs. FCS 5% + 600 nM apelin 83.29 ± 4.961%, *n* = 3, *P < 0.05; diabetic ECs: serum-free 100 ± 4.811% vs. serum-free + 600 nM apelin 114.7 ± 6.733%, *n* = 3, *P < 0.05, Figures 4E,F). SiRNA-mediated silencing of apelin in SMCs prove apelin-dependency of empagliflozin-mediated prevention of SMC proliferation, but not empagliflozin-mediated prevention of SMC migration (Supplementary Figure VI).

## Discussion

Sodium glucose co-transporter 2 inhibition initially developed to improve glycemic control in T2DM not only provides benefits in blood glucose lowering but also exerts cardiovascular advantages. For the first time, we present conclusive evidence that the SGLT2 inhibitor empagliflozin impairs smooth muscle cell proliferation and accelerates endothelial regeneration *in vitro* and prevents neointimal lesion formation and enhances re-endothelialization after vascular injury *in vivo*.

Our observations on the impact of empagliflozin on EC function and endothelial healing confirmed a significantly improved proliferation and migration capacity of diabetic ECs *in vitro* coherent with an accelerated, normalized re-endothelization after electrical denudation of murine carotid arteries in a diabetic mouse model *in vivo*. However, the impact of empagliflozin on non-diabetic EC function did not reach statistical significance, neither *in vitro* nor *in vivo*. This might be the result of a comparable good cellular function of non-diabetic ECs, so that empagliflozin treatment might not be able to further improve the re-endothelization capacity in these cells. In accordance with our findings, empagliflozin has repeatedly been shown to improve endothelial dysfunction and atherogenesis in diabetic rodents during the last few years (11, 14). We could confirm these empagliflozin-mediated effects on EC function in our mouse models for the investigation of neointimal lesion formation and could thus contextualize them with potential treatment strategies for the prevention of restenosis after percutaneous coronary intervention for the first time.

Importantly and in addition to the improvement of EC function, empagliflozin had opposing effects on diabetic as well as non-diabetic human coronary artery SMC function curbing hyperproliferation and hypermigration *in vitro* and preventing neointimal cell proliferation and neointimal lesion formation in response to wire-induced injury of the femoral artery *in vivo*. Conclusive with these results, two recently published studies provide evidence for a reactive oxygen species (ROS)-dependent anti-proliferative effect of empagliflozin on human aortic SMCs *in vitro*, even though seen at different concentrations of empagliflozin (20, 21). A recent Japanese study investigated the combined effects of empagliflozin and the dipeptidyl peptidase-4 (DPP-4) inhibitor linagliptin, another antidiabetic drug, on SMC function and neointima formation. The authors found comparable results with regard to the *in vitro* effects of SGLT2 inhibition on SMC cell count. Conversely to the here presented results, they reported an attenuated neointimal lesion formation in diabetic *db/db* mice following combined treatment with empagliflozin and linagliptin, but not following single treatment with empagliflozin or linagliptin. Remarkably, the guidewire used in this study for endovascular was considerably thinner than the one we have chosen (0.25 mm vs. 0.38 mm) resulting in an apparently smaller neointimal lesion size and thereby concealing the effects of empagliflozin on negative vascular remodeling (22).

Using RNA sequencing (RNA-seq) to decipher further mechanistic details, we identified the vasoactive peptide apelin to be upregulated in diabetic human coronary artery SMCs in response to empagliflozin treatment. Even though apelin did not count to the top regulated genes, it could be found in two of the most significantly regulated gene sets, which implicated apelin in both the negative regulation of SMC proliferation and the improvement of EC function. Apelin is widely expressed in various organs including heart and vasculature and is the endogenous ligand for the APJ receptor, which has been linked to the pathogenesis of cardiovascular diseases (23). The apelin-APJ pathway here appears to have opposing physiological roles to the renin-angiotensin system (24). In ECs, apelin has been shown to induce the release of nitric oxide (NO) and to promote cell proliferation and vascular healing (25–27). Importantly, apelin has also been shown to inhibit the proliferation and migration of murine aortic SMCs (28). As the effect of empagliflozin on SMCs, the apelin-dependent regulation of SMC function has been reported to be mediated through ROS. We could approve the empagliflozin-induced upregulation of apelin in diabetic SMCs as well as in diabetic ECs. Even although some contradictory results on the preventive effects of apelin on SMC proliferation and migration have been published, we could confirm the opposite effects on cell proliferation in diabetic SMCs and ECs treated with recombinant apelin (29–31). EC and SMC interaction has recently gained recognition in the pathogenesis of vascular remodeling processes and a likewise crosstalk through apelin seems to be plausible (32, 33). However, experimental clarification of this thesis goes beyond the scope of this study and might be subject of future studies.

The *in vivo* regulation and effect of apelin in rodent models of vascular remodeling is ambiguous: Global apelin knockout in mice has been shown to prevent neointimal lesion formation in non-diabetic mice (34). Carotid artery balloon injury increased vascular apelin and APJ mRNA expression in Wistar rats *vice versa*. Interestingly, pharmacological prevention of neointimal lesion formation by olmesartan further increased its expression in the same animals (35). In contrast, vascular apelin expression has been shown to be reduced in SMCs of spontaneously hypertensive rats compared to Wistar rats. However, exogenous apelin further increased SMC proliferation rates in this model (36). Finally, apelin prevents the angiotensin II-induced development of atherosclerotic lesions in apolipoprotein E^–/–^ mice (37). All of the aforementioned results reflect the filigree regulation of apelin in vascular remodeling, even though most of the mentioned studies investigated whole vessel samples. Conditional knockout studies might be needed to finally clarify regulation and function of apelin in vascular disease. Even though we did not decipher the consecutive complex signaling cascade, we suppose the effects of empagliflozin on SMCs, ECs, and neointima formation to be at least in part apelin-dependent.

It has been discussed controversially, whether the impact of empagliflozin on cardiovascular outcomes is specifically dependent on myocardial or vascular SGLT2, SGLT2-independent, or the result of a concerted action and simultaneous modulation of multiple molecular and biochemical pathways (38). We here provide evidence for the existence of vascular SGLT2 in human EC and SMC and suppose a direct involvement of vascular SGLT2 rather than a systemic effect of empagliflozin based on the results of our *in vitro* experiments. Furthermore, siRNA-mediated silencing of SGLT2 impaired SMC proliferation and thereby underlined this assumption, even though we did not finally prove a SGLT2-dependency of empagliflozin-mediated vascular effects.

A recently published small open label, single-center, randomized, two-arm clinical trial provides translational insights and supports our here investigated hypothesis in a clinical setting (39). Hashikata and colleagues assigned 28 insufficiently controlled T2DM patients with coronary artery disease planned for DES stenting to receive empagliflozin (*n* = 15, 19 lesions) or other glucose-lowering drugs (*n* = 13, 15 lesions). The primary endpoint, neointimal lesion size at 12 months after cardiovascular intervention assessed by optical coherence tomography (OCT), was significantly reduced in patients receiving empagliflozin compared with patients receiving other glucose-lowering drugs. Despite several limitations (e.g., small number of patients) and even though univariate regression analysis revealed associations of changes in blood pressure and neointimal lesion size indicating indirect effects, this study clearly underpins our hypothesis.

## Conclusion

We here provide evidence that empagliflozin, recently established as a new standard therapy for heart failure with reduced ejection fraction, simultaneously prevents SMC proliferation and augments EC function finally resulting in improved vascular healing and impaired neointimal lesion formation following injury under non-diabetic, but also diabetic conditions. Even although we did not finally clarify the detailed mechanism, the observed effects might be dependent on vascular SGLT2 and involve the vasoactive peptide apelin. Empagliflozin might thus be promising for the prevention of vascular re-narrowing following percutaneous cardiovascular intervention, especially in diabetic patients at high risk for restenosis.

## Data availability statement

The datasets presented in this study can be found in online repositories. The names of the repository/repositories and accession number(s) can be found below: https://www.ncbi.nlm.nih.gov/geo/, GSE189538.

## Ethics statement

The animal study was reviewed and approved by Lower Saxony’s Institutional Committee for Animal Research (Niedersächsisches Landesamt für Verbraucherschutz und Lebensmittelsicherheit, LAVES).

## Author contributions

JD and LB conducted experiments, analyzed the data, and wrote the manuscript. KKa, LK, KKn, and FK conducted experiments, commented, and edited the manuscript. MS, CP, and SK conducted experiments and analyzed the data. HS, J-MD, and JB provided valuable suggestions and comments and edited the manuscript. DS supervised the study, interpreted the data, and wrote the manuscript. All authors contributed to the article and approved the submitted version.
