# Supplementary material for: Empagliflozin prevents neointima formation by impairing smooth muscle cell proliferation and accelerating endothelial regeneration
Source: Front Cardiovasc Med. 2022 Aug 9;9:956041. doi: 10.3389/fcvm.2022.956041 (PMC9396257; doi:10.3389/fcvm.2022.956041)
Supplement: Supplementary file 1 [file Data_Sheet_1.PDF]

Supplementary Figure I

A

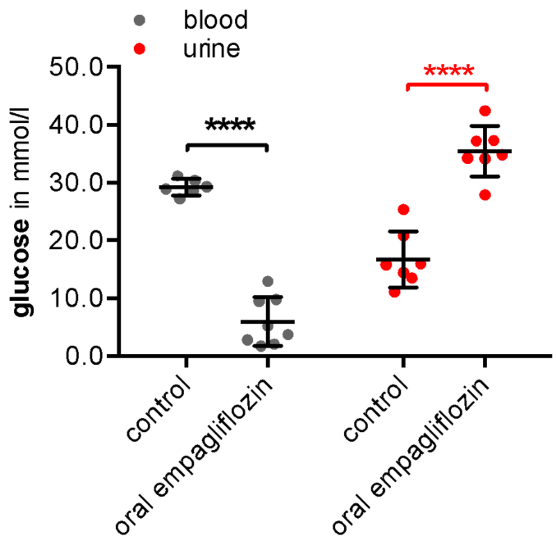

B

	non-diabetic mice		diabetic mice	
	control	empagliflozin	control	empagliflozin
body weight d7	25.9±3.3 g	24.9±1.5 g	28.4±3.0 g	27.4±2.9 g
body weight d28	28.0±1.1 g	24.2±2.2 g	28.4±2.2 g	26.6±3.5 g
Cholesterol	2.2±0.1 mmol/l	2.1±0.3 mmol/l	13.7±1.4 mmol/l	15.7±1.6 mmol/l
HDL	1.7±0.1 mmol/l	1.6±0.2 mmol/l	1.6±0.5 mmol/l	2.7±1.9 mmol/l
LDL	0.23±0.02 mmol/l	0.27±0.02 mmol/l	5.8±0.96 mmol/l	6.31±3.17 mmol/l

Suppl. Fig. I. Empagliflozin reduces blood glucose levels and increases urine glucose concentration, but did not change body weight or lipid levels. Blood and serum glucose concentrations (**a**), body weight at 7 and 28 days, and serum cholesterol concentrations (**b**) measured in mice supplemented with 10 mg kg^{-1} d^{-1} empagliflozin or standard diet (n=6-8, ****P<0.0001, HDL: high density lipoprotein, LDL: low density lipoprotein).

Supplementary Figure II

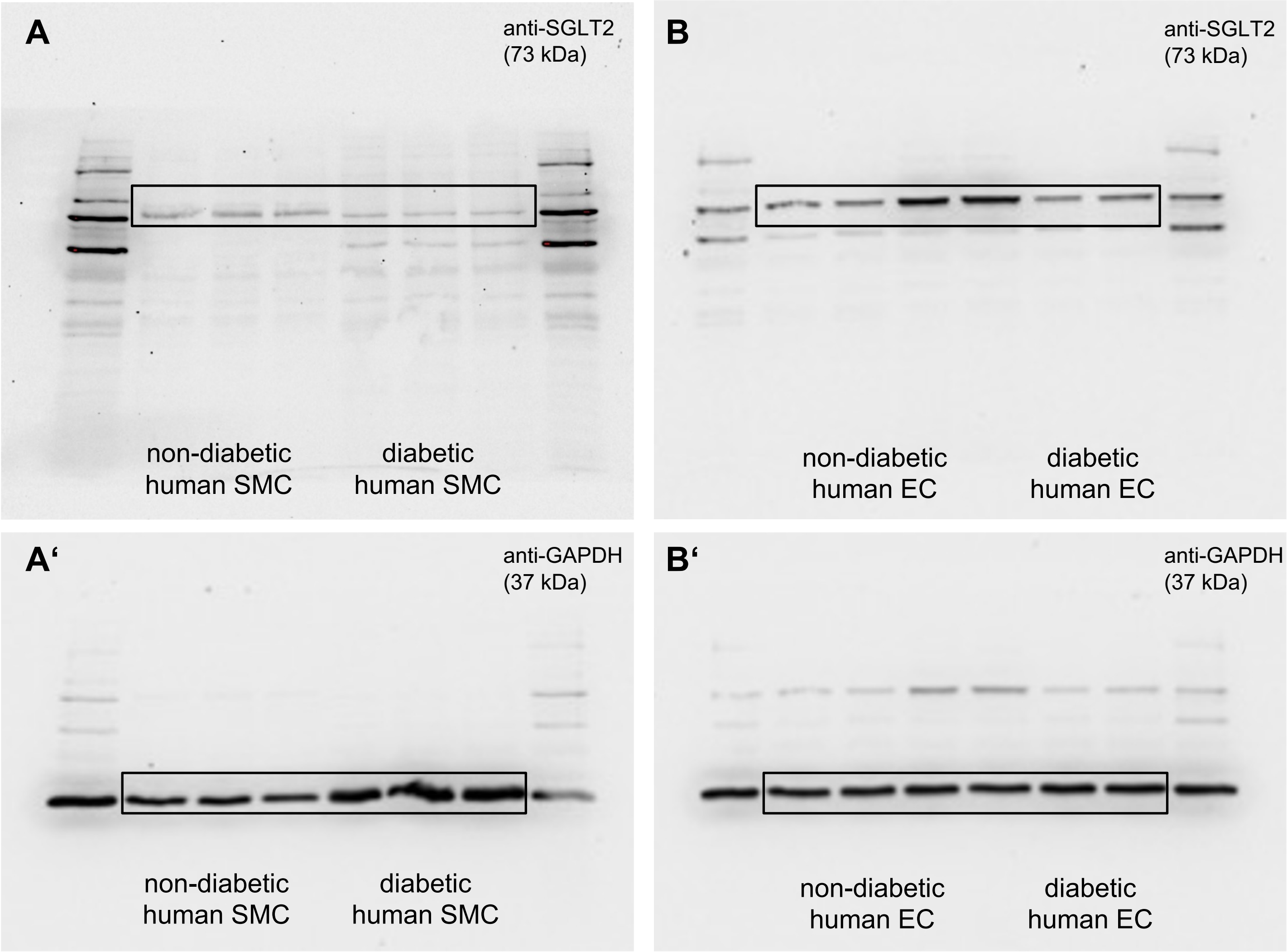

Suppl. Fig. II. SGLT2 is expressed in human vascular cells. Western blot analysis for sodium-glucose co-transporter 2 (SGLT2) protein expression in non-diabetic and diabetic human smooth muscle cells (SMCs, n=3, **a**) and endothelial cells (ECs, n=3, **b**). Glyceraldehyde-3-phosphate dehydrogenase (GAPDH) has been chosen as loading control for the presented blots (SMCs, **a'** and ECs, **b'**)**.** Black boxes mark the cropped parts presented in figure 1.

Supplementary Figure III

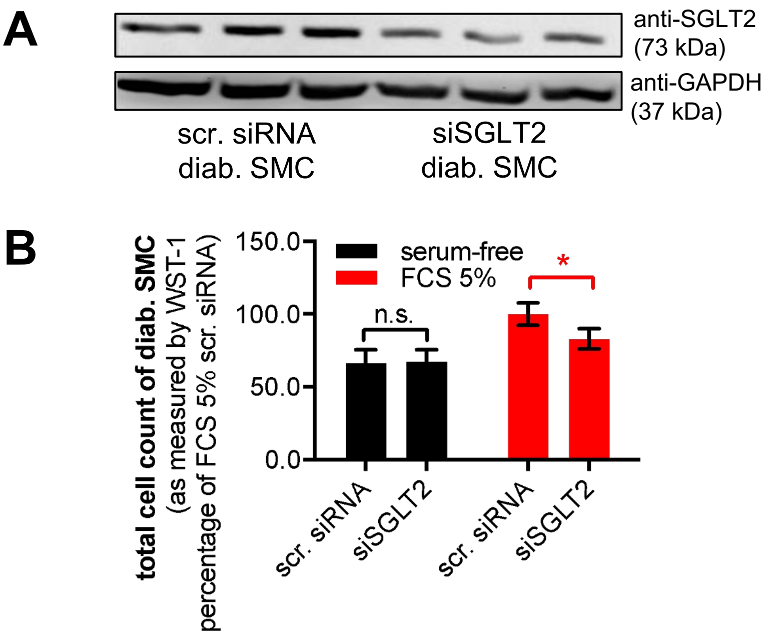

Suppl. Fig. III. Silencing of SGLT2 prevents diabetic smooth muscle cell (SMC) proliferation. a Western blot analysis of SGLT2 protein expression in diabetic SMCs following siRNA-mediated knockdown of SGLT2 (n=3). **b** Total cell count assessment of diabetic SMCs incubated with serum-free medium or 5% FCS with or without siSGLT2 assessed by WST-1 cleavage to formazan at 24h (n=3, *P<0.05).

Supplementary Figure IV

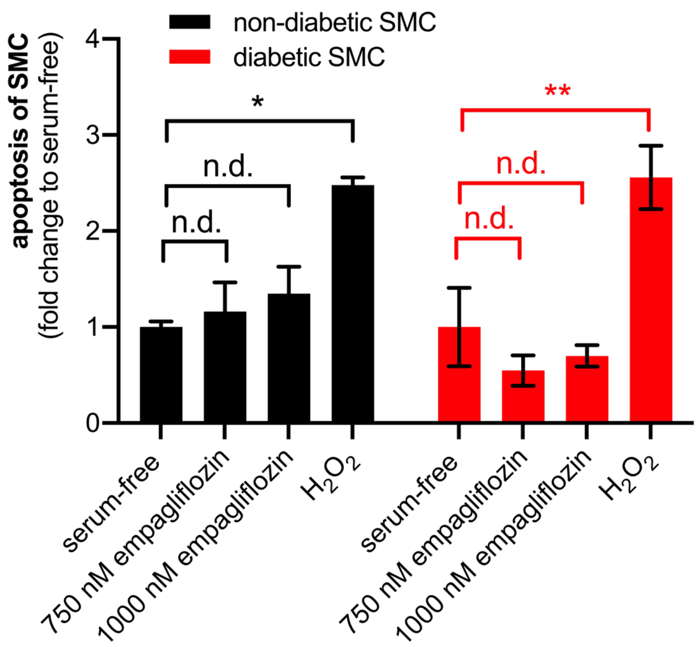

Suppl. Fig. IV. Empagliflozin did not induce SMC apoptosis. Quantification of apoptosis based on the analysis of histone-associated DNA fragments in non-diabetic (black) and diabetic (red) cells treated with indicated concentrations of empagliflozin.

Supplementary Figure V

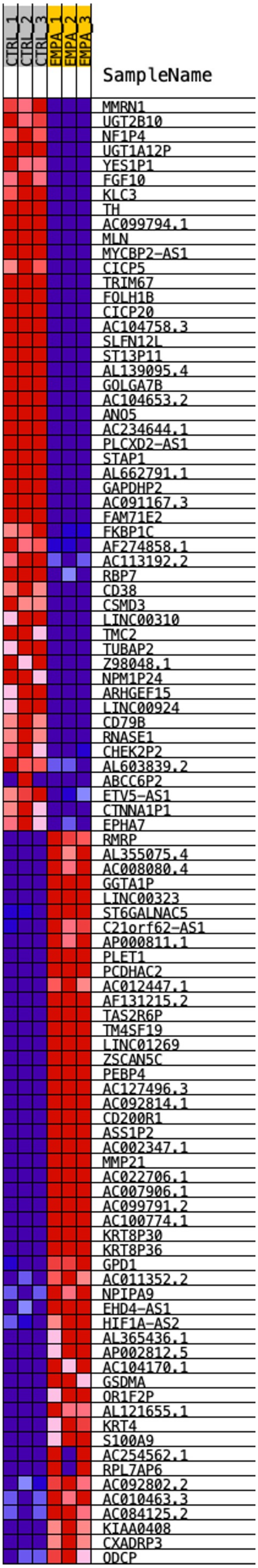

Suppl. Fig. V. Heat map of RNA-sequencing analysis illustrating the expression profile of the top differential expression genes in diabetic smooth muscle cells incubated with (EMPA) or without (CTRL) 750 nM empagliflozin.

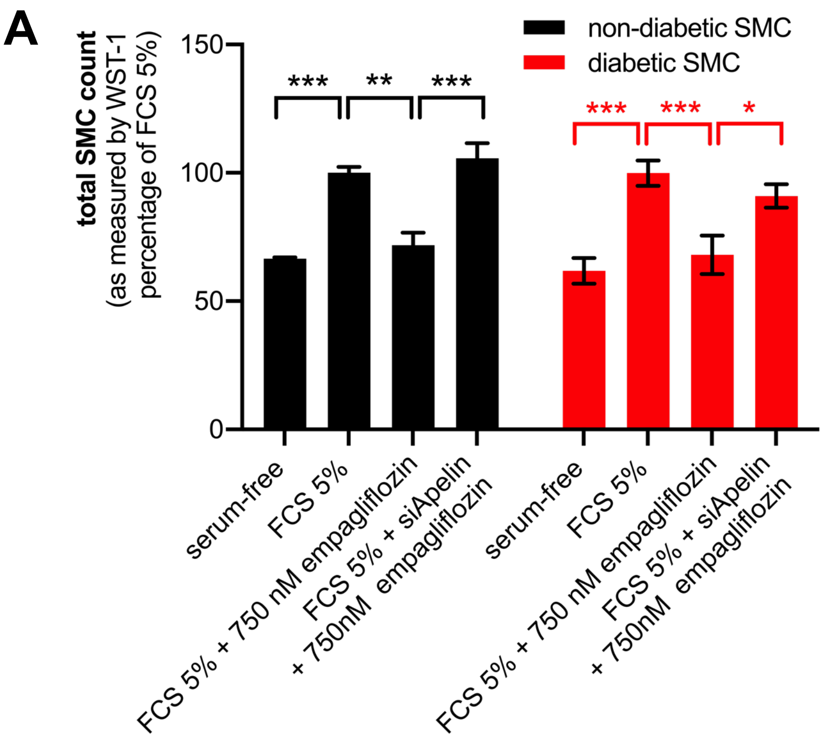

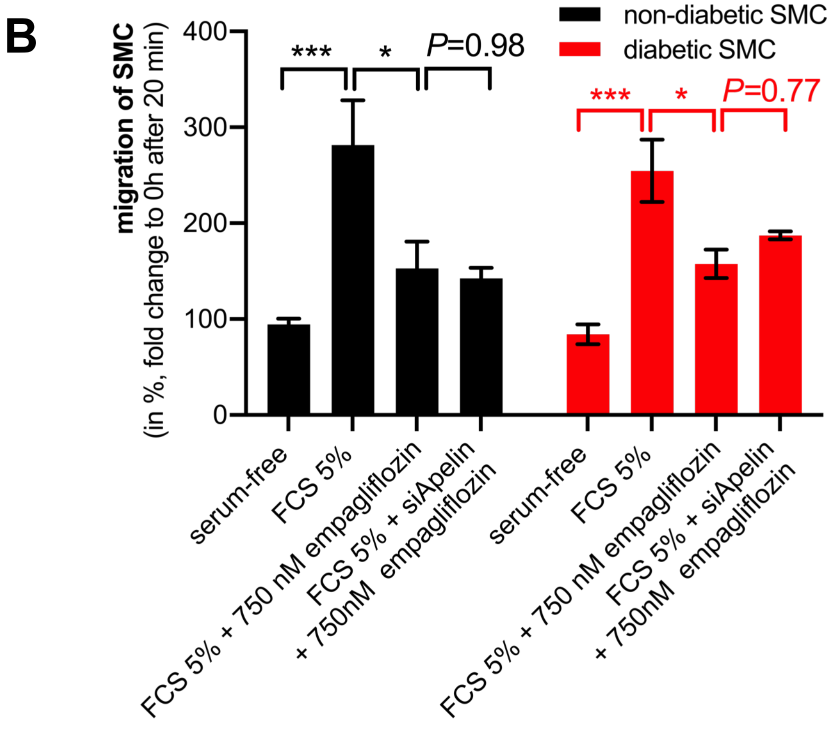

Suppl. Fig. VI. Silencing of apelin prevented empagliflogin-mediated inhibition of SMC proliferation but not SMC migration. Total cell count assessment (a) and assessment of migrational capacity (b) of non-diabetic and diabetic SMCs stimulated with 5% FCS with or without 750 nM empagliflozin transfected with scrambled siRNA (not indicated) or siRNA-mediated silencing of apelin (as indicated). Total cell count has been assessed by WST-1 cleavage to formazan at 24h, cell migration has been assessed by cell coverage 20 minutes in a scratch-wound assay (n=3, *P<0.05, ***P<0.001).
